# Linking egg size, embryonic development, and post-hatch thermoregulatory responses in broilers from young and old breeders

**DOI:** 10.1016/j.psj.2026.106403

**Published:** 2026-01-07

**Authors:** Robson Mateus Freitas Silveira, Sergio Luís de Castro Junior, Felipe Dilelis, Aérica Cirqueira Nazareno, Jumara Coelho Ticiano, Iran José Oliveira da Silva

**Affiliations:** a Livestock Environment Center (NUPEA), Department of Biosystems Engineering, “Luiz de Queiroz” College of Agriculture, University of São Paulo (ESALQ/USP), Av. Pádua Dias, 11 – Agronomia, Piracicaba, SP, 13418-900, Brazil; b Department of Animal Science, “Luiz de Queiroz” College of Agriculture, University of São Paulo (ESALQ/USP), Av. Pádua Dias, 11 – Agronomia, Piracicaba, SP, 13418-900, Brazil; c Grupo Alvorada Incubatório, Itapetininga, São Paulo, Brazil

**Keywords:** Body weight, CHAID decision tree, Egg weight, Respiratory rate

## Abstract

This study evaluated how breeder age influences egg traits, embryonic development, and post-hatch thermoregulatory responses in broilers reared under thermoneutral conditions. Fertile Cobb 500® eggs from 34- and 63-week-old breeders were incubated under standardized conditions in a split-plot design (breeder age × incubation day: 0, 7, 14, and 18 d; *n* = 160). Initial (IW) and final egg weight (FW), embryo weight (EW), ratio between net internal egg weight and embryo weight (NW/E) and embryo length (EL) were recorded. A second experiment reared chicks from the same breeder ages to 42 d in a completely randomized 7 × 2 factorial design (week of age × breeder age; *n* = 48), with weekly measurements of body weight (BW), respiratory rate (RR), cloacal temperature (CT) and surface temperatures (back, head, eye, wing, and foot). Eggs from 63-wk breeders showed greater IW and FW (*p* < 0.001) and higher NW/E (*p* < 0.01). EW and EL increased with incubation day (*p=0.026* and *p=0.004*, respectively), with breeder age × day interactions (EW: *p* = 0.001; EL: *p* = 0.009). Factor analysis consistently yielded two components representing egg size/reserves (IW, FW, and NW/E) and embryonic growth (EW and EL) in both breeder age groups. Post-hatch, RR was influenced by breeder age (*p* = 0.04), week (*p* < 0.001) and their interaction (*p* = 0.001), whereas EyeT showed modest breeder-age effects (*p* = 0.01). CT, HeadT and WingT remained stable (*p>* 0.05). BW was affected only by week (*p* < 0.001), with no effect of breeder age (*p* = 0.95) or interaction (*p* = 0.99). CHAID decision trees highlighted IW (incubation) and BW (post-hatch) as the primary split variables. Practically, breeder age should primarily guide egg grading and incubation management, whereas broilers from young and old breeders can be managed similarly in the grow-out phase under adequate thermal conditions.

## Graphical abstract

**Figure:**
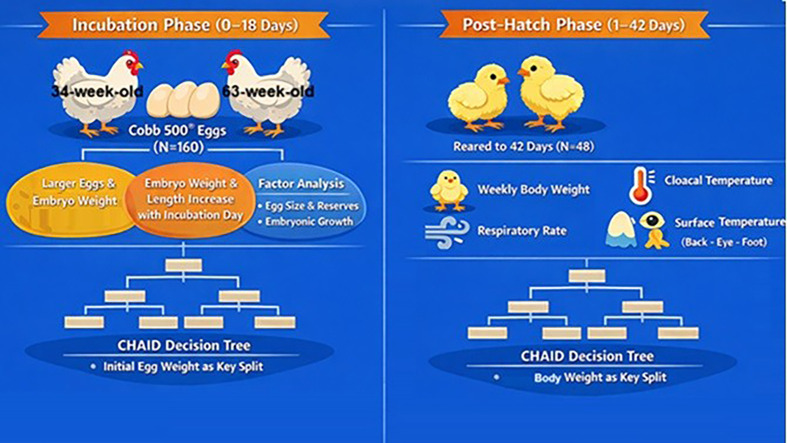

## Introduction

Breeder age markedly influences egg composition and embryonic development (Suarez et al., 1997; Sahan et al., 2014). As breeders age, eggs become larger with more yolk, resulting in heavier embryos at hatch. These differences likely alter the nutrient supply available to the chick and affect early growth and adaptation.

After hatch, chicks rely on absorption of the residual yolk sac for energy during the first week (Ravindran and Abdollahi, 2021). Therefore, variations in embryonic development and hatchling yolk reserves may directly influence early performance and robustness. As a result, differences in embryo growth or yolk reserves due to breeder age could shape chick performance in this critical period.

Chicks also establish homeothermy during the first weeks and are sensitive to heat stress, which can impair growth and welfare (Candido et al., 2020; Brugaletta et al., 2022; Bastaki et al., 2024). However, the link between embryonic conditions (shaped by breeder age and egg traits) and post-hatch thermoregulatory capacity remains largely unexplored, highlighting the novelty of our approach.

In light of this background, we hypothesized that breeder age (34 vs. 63 weeks) modulates thermoregulatory function and performance by altering egg size and embryonic growth. Specifically, chicks from older breeders, having greater yolk reserves and higher body weight at hatch, were expected to maintain a growth advantage through 42 days and exhibit distinct thermoregulatory responses, since heat dissipation capacity is closely linked to body size.

The objective of this study was to simultaneously characterize egg and embryonic traits and evaluate thermoregulatory responses (respiratory rate, cloacal and surface temperatures) and performance in broiler chickens from 0 to 42 days of age, originating from 34- and 63-week-old breeders. This integrative approach aims to clarify how breeder age and early growth jointly shape the relationships between egg and embryo traits and post-hatch thermoregulation and growth

## Materials and methods

### Experiment 1 – Embryonic development

The experiment was conducted at the Livestock Environment Laboratory of the *Núcleo de Pesquisa em Ambiência* (NUPEA), University of São Paulo, "Luiz de Queiroz" College of Agriculture (ESALQ/USP), Piracicaba, SP, Brazil (22°42′30″ S and 47°38′00″ W). All animal procedures were approved by the Institutional Animal Care and Use Committee of ESALQ/USP (protocol n^o^. 8443011024).

Fertile eggs from commercial COBB 500® broiler breeders were obtained from a single farm and collected on the same day. Two breeder ages were evaluated (34 and 63 weeks), representing early/intermediate and late laying stages; 160 eggs were used in the experiment (80 per age), and 50 were kept as reserves. Each egg was individually identified and weighed before incubation.

Eggs were randomly distributed among three forced-draft incubators set at 37.5°C, 55% relative humidity, and automatic turning every 2 h; the room temperature was maintained at 25°C. Air temperature and relative humidity were monitored at the geometric center of each incubator using data loggers (Onset HOBO U12-013) recording at 5-min intervals. Incubators remained closed except during sampling.

Random samples (*n* = 20 eggs per breeder age) were removed on days 0, 7, 14, and 18 of incubation. Immediately after removal, eggs were candled in a dark room to identify infertile eggs or embryonic mortality; when necessary, these were replaced with reserve eggs of similar appearance and developmental stage, according to the COBB incubation management guide.

### Egg traits and embryonic development

Initial and final egg weights were recorded at the time of removal from the incubator using a high-precision digital scale (Gehaka, BG 2000) to estimate egg weight loss during incubation.

At each sampling time, fertile eggs were carefully opened and the embryo and associated structures were removed. Embryo weight and residual internal fluid were recorded to estimate the relative contribution of the embryo to total egg weight. Embryo length was measured using a digital caliper (Digimess, 100.174BL). The procedures adopted in this phase were adapted from the methodologies described by Chan and Burggren (2005) and Donofre (2018).

### Experiment 2 – Post-hatch period – 0 to 42 days

#### Experimental site and ethical approval

The experiment was conducted in a Climate-controlled Chamber at ESALQ/USP. All procedures were approved by the Institutional Animal Care and Use Committee (CEUA) of ESALQ/USP, under protocol n^o^. 1033260924

### Animals

A total of 48 Cobb commercial broiler chickens were used, with an initial average body weight of 43.8 ± 2.47 g and a final average body weight of 2524 ± 284 g. Birds were housed on rice-straw litter, with *ad libitum* access to water and feed. Experimental diets were formulated according to the recommendations of the strain management guide, and the chemical composition of the ingredients followed the tables of Rostagno et al. (2017).

### Housing

The experiment was carried out in a climatic chamber divided into two pens to facilitate handling. The pens were built with pine wood and galvanized wire mesh, each measuring 1.50 m in length, 1.00 m in width and 0.70 m in height, resulting in a usable floor area of 1.5 m² per pen. Birds were allocated to achieve a stocking density of 12 birds/m², in accordance with common experimental practice for broilers.

Microclimatic variables (air temperature and relative humidity) were controlled by the climatic chamber itself and monitored using two Hobo® dataloggers (model U10–003, Onset Computer Corporation, MA, USA), placed in each pen and programmed for continuous recording. Wind velocity and light intensity were monitored using an anemometer (AK835, Akso; range 0.4–30.0 m/s, accuracy ±3% + 0.1 unit) and a digital lux meter (INS-1381, Metrins; range 0–200,000 lux), respectively. Air temperature, relative humidity, ventilation and lighting program were adjusted to meet the thermoneutral and management requirements of the strain, according to the Cobb Management Guide, including the specific lighting schedule recommended for each rearing phase.

### Data collection

Physiological and performance data were collected weekly at 0, 7, 14, 21, 28, 35 and 42 days of age. At each of these ages, birds were evaluated individually. Body weight (BW) was measured using a digital precision scale, and these values were used to describe the growth curve over the experimental period and to integrate subsequent analyses with physiological variables.

Respiratory rate (RR, breaths min⁻¹) was obtained by counting thoracic movements for 15 seconds and multiplying the recorded value by four. Cloacal temperature (CT,°C) was measured with a digital thermometer (accuracy ±0.1°C, G-Tech, Rio de Janeiro, Brazil), inserted approximately 3 cm into the cloaca until the reading stabilized. Surface temperatures of different body regions (back, head, eye, wing and foot) were measured using an infrared thermometer (accuracy ±0.1°C, STHT 77365, Stanley, USA), positioned at a distance of about 15 cm from the bird’s body.

### Statistical analysis

#### Experiment 1 – Embryonic development

The response variables associated with egg and embryo traits were treated as continuous quantitative measures. The experiment followed a split-plot design, in which breeder age (34 and 63 weeks) was assigned to the main plots and incubation day (0, 7, 14 and 18 days) to the subplots. The statistical model used for the analysis of these data is presented below.

(1) $$y_{ijk}=\mu +\alpha_{i}+e_{ij}+\beta_{k}+{\left(\alpha \beta \right)}_{ik}+\epsilon_{ijk}$$

In which:

y_ijk_ represents the observed value of the response variable in the j^th^ egg (replication), belonging to the i^th^ level of the hen age factor, on the k^th^ day of incubation;
μ is the overall mean;
α_i_ is the fixed effect of hen’s age;
e_ij_ is the random error associated with the plot (matrix);
β_k_ is the fixed effect of incubation days;
(αβ)_ik_ represents the interaction between hen age and incubation days;
ϵ_ijk_ is the random error associated with the subplots. The indices vary as follows: *i* = 34, 63 (weeks); *j* = 1, 2, ..., 20; *k* = 0, 7, 14, 18 (days).

The statistical model was used to analyze egg-related variables and embryonic physical traits, assuming residual normality and homoscedasticity. In the absence of a significant interaction between factors, main-effect means were compared independently. When a significant age × incubation day interaction was detected, the analysis was decomposed, assessing the effect of one factor within each level of the other. Multiple comparisons of means were carried out using Tukey’s test at a 5% significance level.

Whenever violations of the model assumptions (normality and/or homoscedasticity) were identified, a Box–Cox transformation (Box and Cox, 1964) was applied as a corrective procedure. In situations where the transformation did not adequately stabilize the variance, particularly in the presence of marked skewness or outliers, Generalized Linear Models (GLM) were fitted, following the framework of Nelder and Wedderburn (1972) and McCullagh and Nelder (1989), assuming gamma or inverse-Gaussian distributions, according to best fit. Model selection was based on likelihood ratio tests and visual inspection of half-normal plots (Moral et al., 2017). All statistical analyses were performed using R software (R Core Team, 2023).

#### Experiment 2 – Post-hatch period – 0 to 42 days

The response variables related to physiological traits and performance were treated as continuous quantitative measures. The experimental design was completely randomized in a 7 × 2 factorial arrangement, with seven broiler ages (1 to 7 weeks) and two breeder ages (34 and 63 weeks) as fixed factors. Each bird was considered an experimental unit. The analysis was conducted based on the following general linear model:

Y_ijk_ =μ +G_i_ + S_j_ + (G_i_ × S_j_) + e_ijk_

In which:

Y_ijk_ represents the observed response variable for the k-th replicate of the ith broiler age and j-th breeder age;
µ is the overall mean;
G_i_ represents the effect of the ith broiler age (*i* = 1, 2, 3, 4, 5, 6, and 7 weeks);
S_j_ represents the interaction effect between broiler age and breeder age;
G_i_ × S_j_ corresponds to the interaction effect of breeder age and broiler age
e_ijk_ is the random error associated with each observation.

Initially, a two-way factorial ANOVA model was fitted, including the main effects of breeder age, broiler age (week), and their interaction (breeder age × week). Model adequacy was assessed by graphical inspection of the residuals (residuals vs. fitted values and QQ-plots) and by formal tests of normality and homogeneity of variances. Homoscedasticity among groups was specifically evaluated using Levene’s test. Since all assumptions of residual normality and variance homogeneity were met, there was no need for data transformation or the use of generalized linear models.

In addition, a post hoc power analysis was performed, considering the observed effect sizes and the adopted significance level, in order to verify the probability of detecting true effects for the main contrasts of interest. In the absence of a significant interaction, main effects were interpreted directly and means were compared using Tukey’s test. When a significant interaction was present, the interaction was decomposed, and the effect of one factor was evaluated within each level of the other. A significance level of 5% (α = 0.05) was adopted

### Multivariate analyses and machine learning approach

Exploratory factor analysis (EFA) was applied separately to the incubation and post-hatch datasets. In the incubation phase, the correlation matrix among egg and embryo variables was used, with analyses conducted independently for progeny from 34- and 63-week-old breeders. Similarly, in the post-hatch phase, EFA was applied to physiological and performance variables, again in separate analyses by breeder age. Factor analysis was conducted using the standard factor model

$$X_{1}=a_{11}\times F_{1}+\;a_{12}\times F_{2}+\cdots +\;a_{1m}\times F_{m}+\;e_{p}$$

$$\begin{array}{c} X_{2}=a_{21}\times F_{2}+\;a_{21}\times F_{2}+\cdots +\;a_{2m}\times F_{m}+\;e_{p}\\ \vdots \end{array}$$

$$X_{p}=a_{p1}\times F_{1}+\;a_{p1}\times F_{2}+\cdots +\;a_{pm}\times F_{m}+\;e_{p}$$

In which:

$X_{p}\;$is the p ^th^ score of the standardized variable (*p* = 1, 2, …, m)
$F_{m}\;$is the extracted factor, $a_{pm}$is the factor loading, and $e_{p}$is the error.

Factor scores for each group were estimated by multiplying standardized variables by the coefficient of the corresponding factor score, as follows:

$$F_{1}=d_{11}\times X_{1}+\;d_{12}\times X_{2}+\;\cdots +\;d_{1j}\times X_{jp}$$

$$\begin{array}{c} F_{2}=d_{21}\times X_{2}+\;d_{21}\times X_{2}+\cdots +\;d_{2j}\times X_{jp}\\ \vdots \end{array}$$

$$F_{j}=d_{p1}\times X_{1}+\;a_{j1}\times X_{2\;}+\;\cdots +\;d_{jp}\times X_{jp}$$

In which:

$F_{j}\;$is the j- ^th^ factor extracted,
$d_{pj}\;$is the factor score coefficient
p is the number of variables.

In both phases, data suitability for factor analysis was assessed using the Kaiser–Meyer–Olkin (KMO) index, adopting values > 0.60 for all variables as the minimum adequacy criterion, and Bartlett’s test of sphericity, considering *p* < 0.001 as evidence of a sufficient correlational structure to justify dimensionality reduction. Factors were initially extracted using the principal components method, retaining those with eigenvalues greater than 1.0, complemented by visual inspection of the scree plot. To facilitate interpretation of the latent dimensions, varimax orthogonal rotation was applied, and factor loadings with an absolute value ≥ 0.40 were deemed relevant. Based on the rotated loadings, factors were interpreted in terms of physiological and productive axes (for example, “egg size/reserves” vs. “embryonic growth” in the incubation phase; “body size/peripheral temperature” vs. “regional temperature distribution” in the post-hatch phase), and, when appropriate, factor scores were computed for use in biplots and in the discussion of multivariate profiles.

Canonical discriminant analysis was used to evaluate the ability of sets of continuous variables to discriminate among groups defined by breeder age × time. The general CDA model is described below.

$$Z_{n}=\;\propto +\;\beta_{1}X_{1}+\beta_{2}X_{2}+\;\cdots +\;\beta_{n}X_{n}$$

In which:

$Z_{n}$ represents the canonical discriminant function (or canonical variable), defined as a linear combination of the original variables used to discriminate among groups,
∝is the intercept,
$X_{i}$are the explanatory variables,
$\beta_{i}$are the discriminant coefficients for each explanatory variable.

All variables were standardized beforehand (zero mean and unit variance) to remove scale effects. In the incubation phase, the grouping variable was the combination of breeder age (34 and 63 weeks) and incubation day (0, 7, 14, and 18 days), resulting in eight groups, and the predictors included egg and embryo measurements. In the post-hatch phase, the grouping variable was the combination of breeder age and week of age (1 to 7 weeks), totaling 14 groups, and BW plus thermal variables were used as predictors. In both phases, a stepwise procedure was applied for variable selection, using reduction in Wilks’ lambda and a 5% significance level as criteria for entry and retention in the model. Canonical functions were evaluated in terms of eigenvalues, canonical correlations, and the proportion of discriminant variance explained, and discriminative ability was quantified using the classification matrix, with calculation of the percentage of correctly classified cases and analysis of misclassification patterns between adjacent time points and breeder ages.

As a complementary classification approach, decision trees were fitted using the CHAID algorithm for both incubation and post-hatch data. In both phases, the response variable was the combined breeder age × time category (incubation day or week of age), and the explanatory variables corresponded to the traits of interest in each phase (egg/embryo traits in the incubation phase and physiological responses plus BW in the post-hatch phase. For tree fitting, the dataset was randomly split into two subsets: 80% of the observations were used to train the model and 20% were reserved for validation, allowing assessment of predictive performance on data not used for model fitting.

## Results

### Incubation phase (embryonic development)

During the incubation phase, egg and embryonic traits were clearly affected by breeder age and incubation day (Table 1). Initial egg weight (IW) ranged from 60.1 to 75.2 g, with an average of 66.95 g, and was consistently greater in eggs from 63-week-old breeders than in those from 34-week-old breeders (*p* < 0.001). Similarly, final egg weight (FW) averaged 63.93 g and was also higher in eggs from the older breeders (*p* < 0.001). In addition, FW declined progressively during incubation (0 > 7 > 14 > 18 d; *p* < 0.001), reflecting loss of mass due to water evaporation and utilization of yolk and albumen reserves.

**Table 1:** Descriptive statistics and ANOVA results for the effects of age (A), week (W) and their interaction (*A* × *W*) on variables related to embryonic development (1–18 d) and the post-hatching period (1–7 wk).

	Descriptive statistics					Significant difference		*p-*value		
	Variables	Minimum	Maximum	Mean	SEM	Age (a)	Day (d)/Week (wk)	Age (A)	Week (W)	A*W
Embryonic Development (0 to 18 d)	IW	60.09	75.22	66.95	0.34	63 > 34 a	-	<0.001	= 0.863	= 0.999
	FW	54.24	75.22	63.93	0.38	63 > 34 a	0 > 7 > 14>18d	<0.001	<0.001	= 0.983
	EW	0.00	37.98	12.16	1.07	-	-	= 0.026	<0.001	= 0.001
	EL	0.00	82.10	38.28	2.35	-	-	= 0.004	<0.01	= 0.009
	NW/E	46.16	62.82	54.97	0.33	63 > 34 a	7 and 14 > 18 d	<0.001	= 0.004	= 0.911
After hatching (1 to 7 wk)	RR	42.00	138.00	80.09	0.95	-	-	= 0.04	<0.001	=0.001
	CT	36.80	42.80	41.07	0.06	-	-	= 0.42	= 0.88	= 0.58
	BackT	28.30	44.70	34.07	0.12	-	1-4 > 6 and 7 wk	= 0.61	<0.001	= 0.43
	HeadT	27.10	43.50	33.97	0.10	-	-	= 0.17	= 0.30	= 0.49
	EyeT	28.70	44.80	34.71	0.12	63 > 34 a	5 to 7 > 1 to 4 wk	= 0.01	<0.001	= 0.05
	PawT	26.10	39.00	31.01	0.17	-	6 and 7 > 1 to 5 wk	= 0.09	<0.001	= 0.46
	WingT	28.40	33.80	33.48	0.10	-	-	=0.22	= 0.16	= 0.27

IW (Initial Weight, g), FW (Final Weight, g), EW (Embryo Weight, g), NW/E (Ratio between net internal egg weight and embryo weight g/g), EL (Embryo Length, mm), RR (Respiratory Rate, breaths·min⁻¹), CT (Cloacal Temperature,°C), BackT (Back Temperature,°C), HeadT (Head Temperature,°C), EyeT (Eye Temperature,°C), PawT (Paw Temperature,°C), WingT (Wing Temperature,°C).

Significant differences between ages or weeks were identified using Tukey’s test (*p* < 0.05).

Traits directly related to embryonic development showed strong effects of both breeder age and incubation day. Embryo weight (EW) and embryo length (EL) showed significant interactions (*p* = 0.001 and *p* = 0.009, respectively) as incubation progressed and with breeder age (Table 1, Table 2). In general, embryos originating from 63-week-old breeders were slightly heavier and longer at the end of incubation; however, the magnitude of these differences varied with incubation day. Ratio between net internal egg weight and embryo weight (NW/E), was also higher in eggs from older breeders (*p* < 0.001) and increased over the incubation period, but showed lower values at day 18 compared with days 7 and 14 (*p* = 0.004), indicating a temporal pattern of embryonic mass accumulation.

**Table 2:** Breakdown of breeder age × time interactions for respiratory rate (weeks), embryo weight and embryo length (incubation days).

Variables	Week	Age	
		63 wk	34 wk
Respiratory rate (breaths·min⁻¹)	1	90.00^aA^	81.00^bB^
	2	80.73^bB^	95.50^aA^
	3	83.40^aAB^	89.25^aB^
	4	85.58^aAB^	87.60^aB^
	5	76.74^aB^	76.88^ªC^
	6	66.86^aC^	68.57^aD^
	7	61.06^aD^	67.88^aD^
	Days		
Embryo weight (g)	0	0.0^aD^	0.00^aD^
	7	1.10ª^C^	0.99^ªC^
	14	13.60ª^B^	13.80ª^B^
	18	33.80^aA^	31.90^bA^
Embryo length (mm)	0	0.00^aD^	0.00^aD^
	7	21.80^aC^	22.11^aC^
	14	55.39^aB^	54.73^aB^
	18	77.12^aA^	72.66^bA^

Different lowercase superscript letters in the same row and uppercase letters in the same column indicate significant differences among incubation days (*p* < 0.05).

EFA applied to egg and embryo traits, conducted separately for progeny from 34- and 63-week-old breeders (Fig. 2), revealed a very similar structure between breeder ages. In both groups, two principal components explained more than 94% of the total variance (Axis 1 ≈ 51%; Axis 2 ≈ 43%). Component 1 was defined by high positive loadings for IW, FW and NW/E, and therefore represented an axis of “egg size and initial reserves”. Component 2 was formed mainly by EW and EL, characterizing an axis of “embryonic development”.

Canonical discriminant analysis of embryonic traits identified IW, EW, and EL as the main variables differentiating embryonic stages, demonstrating a strong ability to separate groups defined by the combination of breeder age and incubation day (Fig. 4). All three canonical functions were significant (*p* < 0.001). Standardized canonical coefficients indicated that Function 1 (93.8%) was dominated by EW (0.750) and EL (0.542), with a modest negative contribution of IW (–0.131). Function 2 (4.9%) was defined mainly by a contrast between EL (0.866) and EW (–0.721).

In the canonical biplot, group centroids were distributed along Function 1 in accordance with incubation progress: eggs evaluated at day 0 clustered at the negative extreme, those at 7 d occupied intermediate scores, and those at 14 and 18 d were located at the most positive scores, regardless of breeder age. Along Function 2, additional separation between progeny of 34- and 63-week-old breeders within the same day was observed, especially at 14 and 18 d, reinforcing that subtle differences in the trajectory of embryonic growth depend on breeder age. The classification matrix confirmed the strong discriminant ability of the model: 85.9% of original cases were correctly assigned to their breeder-age × day group, with misclassifications occurring mainly between the two breeder ages within the same day rather than among consecutive days.

A decision-tree classification analysis applied to the same embryonic dataset further emphasized the central role of IW in distinguishing among breeder-age × day groups (Fig. 5). Considering eight categories, the root node showed a uniform distribution of categories (12.5% for each group). The first—and only—split was performed by IW (adjusted *p* < 0.001; χ² = 89.167), with a cut-off at 64.66 g. Node 1 (IW ≤ 64.66 g) contained mainly eggs and embryos from 34-week-old breeders, regardless of incubation day, whereas categories corresponding to 63-week-old breeders contributed only 1.6–0.0% of cases. Conversely, Node 2 (IW > 64.66 g) was dominated by progeny from 63-week-old breeders, which accounted for about 20% of the records at each day, with a much smaller participation of 34-week categories.

### Post-hatch period (thermoregulatory responses and performance) – 0 to 42 days

Respiratory rate (RR) was the most responsive variable, being affected by breeder age (*p* = 0.04), week (*p* < 0.001) and their interaction (*p* = 0.001). Progeny from 63-week-old breeders showed a progressive decline in RR across weeks, whereas chicks from 34-week-old breeders exhibited an early peak (2 week) followed by a gradual reduction, resulting in slightly higher mean RR overall.

Among surface temperatures, back temperature was influenced only by week (*p* < 0.001), increasing until week 4 and declining thereafter, with no effect of breeder age or interaction (*p* = 0.43). Eye temperature was affected by breeder age (*p* = 0.01) and week (*p* < 0.001), with a trend for an age × week interaction (*p* = 0.05). Chicks from older breeders showed higher eye temperatures and a progressive increase over time (5 to 7 wk). Paw temperature (PawT) was strongly affected by week (*p* < 0.001) and showed a trend for an age effect (*p* = 0.09). In both age groups, PawT increased from 28.9°C in week 1 to 34.0°C in week 7, with no age × week interaction (*p* = 0.46), reflecting a consistent increase in peripheral temperature as birds grew. By contrast, CT, HeadT and WingT remained stable (*p* > 0.05), with CT close to 42°C throughout the period, indicating maintenance of central homeothermy and greater stability of these thermal variables.

**Fig. 1:**
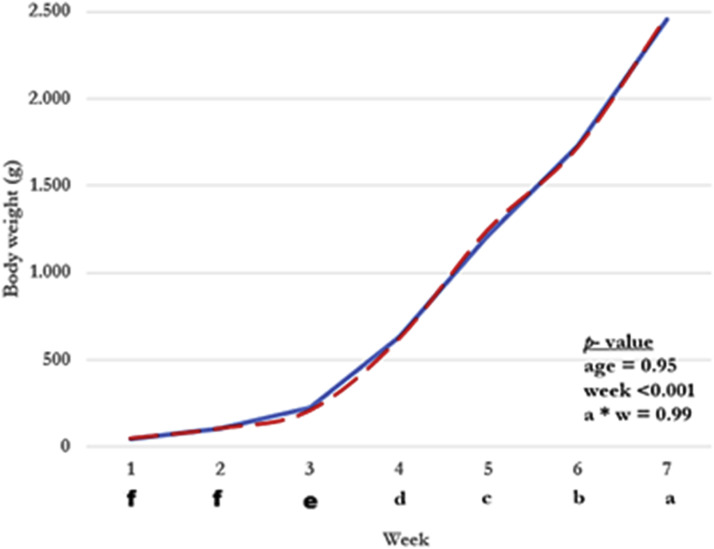
Body weight of broilers from 34- and 63-week-old breeder hens from 1 to 7 weeks of age. *Note:* • Mean body weight (g) of offspring from 34-week-old (solid blue line) and 63-week-old (dashed red line) breeder hens during the growing period (1–7 weeks). • Different lowercase letters indicate significant differences among weeks (*p* < 0.05). • There was no main effect of breeder age (*p* = 0.95) and no age × week interaction (*p* = 0.99), whereas the effect of week was highly significant (*p* < 0.001).

Body weight (BW) of broilers over the seven-week period (Fig. 1) was strongly influenced only by week (*p* < 0.001). There was no effect of breeder age (*p* = 0.95) or of the age × week interaction (*p* = 0.99), and the growth curves of both breeder-age groups were almost superimposed. A marked increase in BW occurred from week 3 onward, with each subsequent week differing from the previous one until the highest weights were reached in week 7, characterizing an approximately linear growth pattern.

**Fig. 2:**
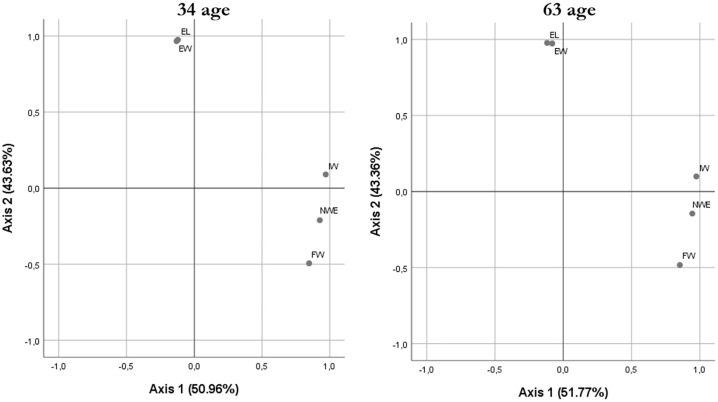
Factor-analysis biplot of egg and embryo traits in progeny from 34- and 63-week-old breeders. IW (Initial Weight, g), FW (Final Weight, g), EW (Embryo Weight, g), NW/E (Ratio between net internal egg weight and embryo weight, g/g), EL (Embryo Length, mm).

**Fig. 3:**
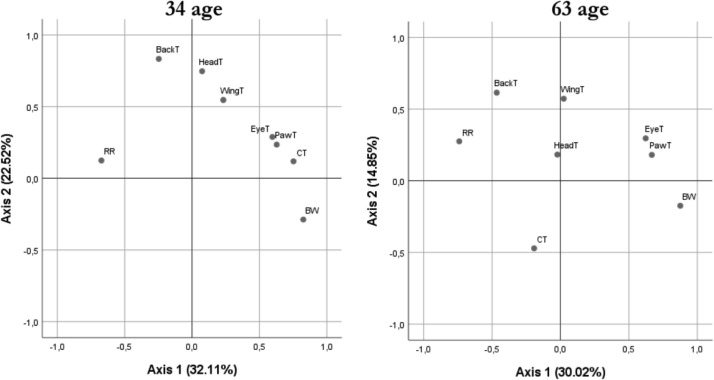
Factor-analysis biplot of physiological and productive responses of broilers from 34- and 63-week-old breeders. RR (Respiratory Rate, breaths·min⁻¹), CT (Cloacal Temperature,°C), BackT (Back Temperature,°C), HeadT (Head Temperature,°C), EyeT (Eye Temperature,°C), PawT (Paw Temperature,°C), WingT (Wing Temperature,°C).

EFA of post-hatch physiological responses and performance, conducted separately for progeny from 34- and 63-week-old breeders (Fig. 3), showed that in birds from 34-week-old breeders, Component 1 explained 32.11% of the variance and was defined by positive loadings for EyeT, CT and PawT and a negative loading for RR, representing a gradient contrasting higher body size and surface temperatures with lower respiratory rate. Component 2 (22.52%) was associated mainly with BackT and HeadT. In progeny from 63-week-old breeders, Component 1 accounted for 30.02% of the variance and grouped EyeT and PawT with positive loadings and CT and BW with a negative loading. Component 2 (14.85%) was mainly associated with BackT, WingT, and RR, resembling the pattern observed in progeny from 34-week-old breeders.

**Fig. 4:**
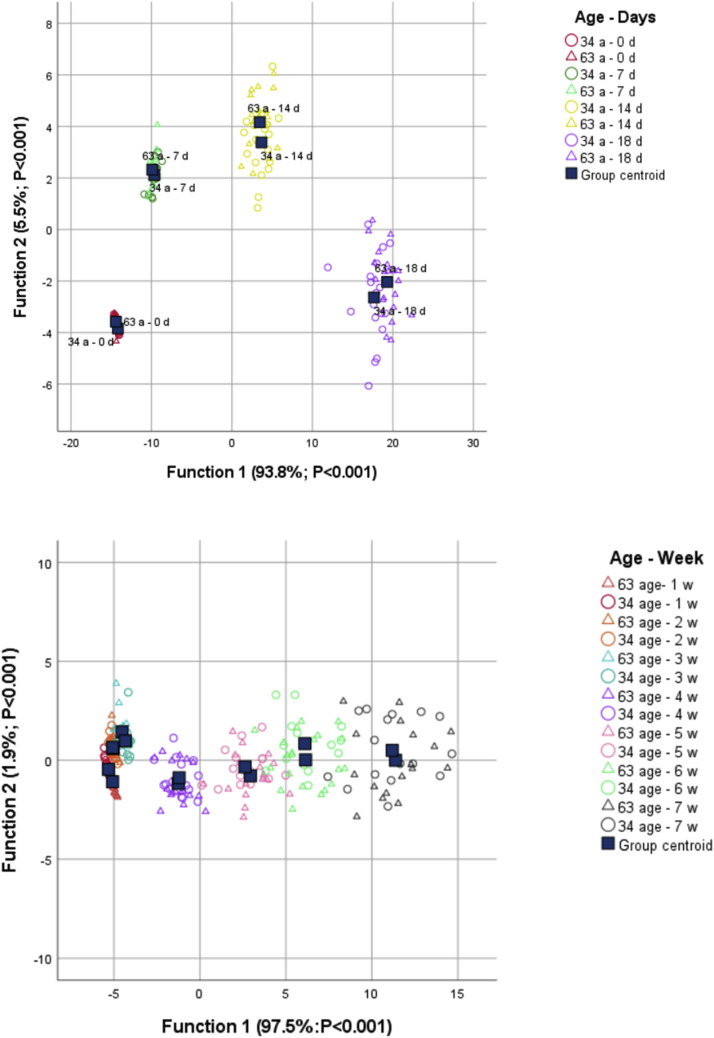
Canonical discriminant analysis of embryonic development and post-hatch of broilers from 34- and 63-week-old breeders.

Canonical discriminant analysis using BW, RR, CT and surface temperatures revealed three significant canonical functions (Fig. 4). Function 1 had an eigenvalue of 35.675 and accounted for 97.5% of the total discriminant variance, with a canonical correlation of 0.986, and was dominated by BW (standardized coefficient = 1.041), with small negative contributions from EyeT (–0.170) and PawT (–0.105). Thus, the main discriminant gradient among groups defined by breeder age × broiler age was driven by changes in body weight. Function 2 explained an additional 1.9% of the variance and was defined mainly by PawT (0.907) and EyeT (–0.433), representing a thermal gradient contrasting higher foot temperature with higher eye temperature. The canonical biplot showed a progressive shift of group centroids along Function 1 with increasing bird age, whereas Function 2 provided additional separation between progeny of 34- and 63-week-old breeders at intermediate ages. The classification matrix indicated an overall accuracy of 59.9%, with most misclassifications occurring between breeder ages within the same week and, in older birds, between adjacent weeks, suggesting a continuum of growth and thermoregulatory development rather than sharp boundaries among age classes.

**Fig. 5:**
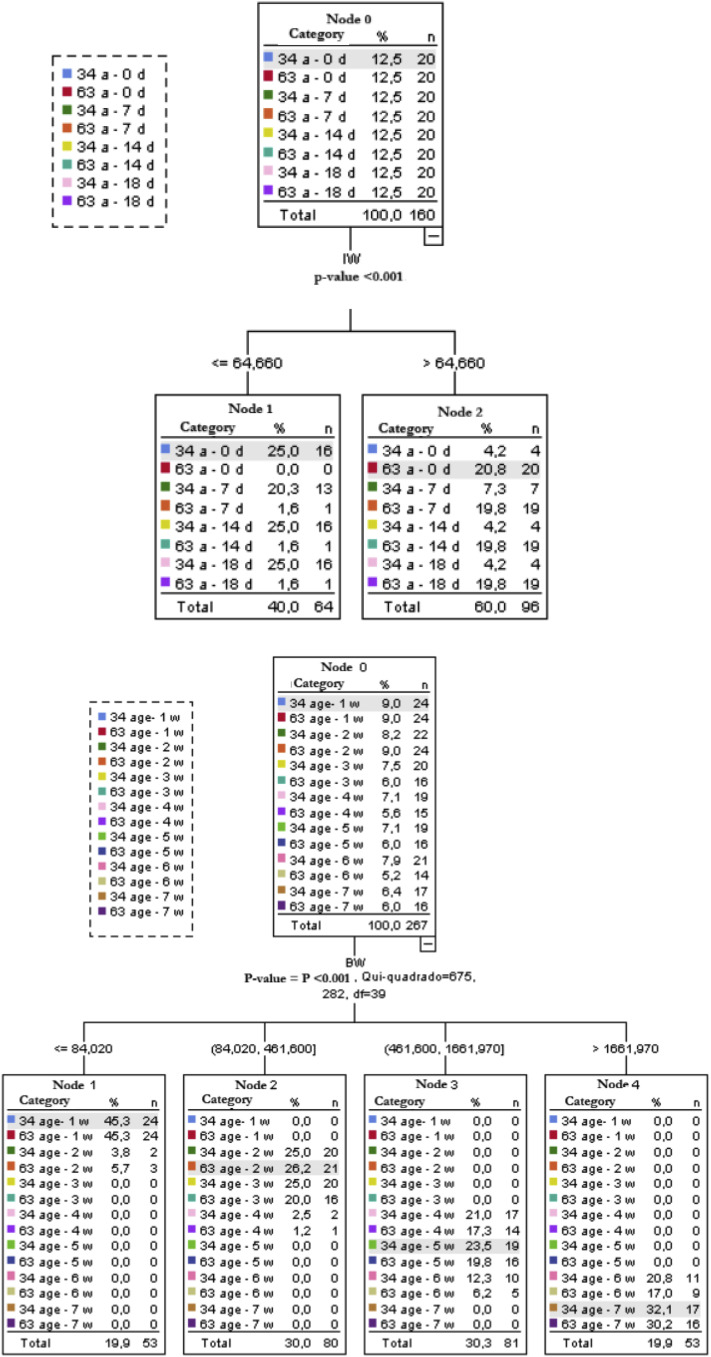
Decision Tree of embryonic development and post-hatch of broilers from 34- and 63-week-old breeders.

Complementary decision-tree analysis also highlighted BW as the main predictor for discriminating the combined breeder-age × week categories (age_Week; χ² = 675.282; Fig. 5). The root node grouped the 14 age–week combinations with similar frequencies (approximately 5–9% of records per category). The first split occurred in four BW ranges (≤ 84.02, 84.02–461.60, 461.60–1,661.97 and > 1,661.97 g). The lowest BW range (≤ 84.02 g) contained mainly birds in the first week from both breeder ages (45.3% of each group, with a small contribution from week 2). The low–intermediate range (84.02–461.60 g) comprised almost exclusively birds in weeks 2 and 3 at both ages (each breeder-age × week combination contributing about 20–26% of the cases). The high–intermediate range (461.60–1,661.97 g) concentrated birds in weeks 4, 5 and 6 (approximately 17–24% for each combination), whereas the highest BW range (> 1,661.97 g) included mainly birds in weeks 6 and 7 (20.8% and 17.0% for progeny of 34- and 63-week-old breeders in week 6, and 32.1% and 30.2% in week 7, respectively).

## Discussion

### Breeder age and embryonic development

Our data demonstrate that breeder age affects development from the embryonic stage onward, as eggs from older breeders (63 weeks) were significantly larger and produced heavier and longer embryos at advanced stages of incubation. These findings are consistent with previous studies reporting increases in egg weight and yolk content with advancing breeder age, resulting in heavier embryos and chicks (Nangsuay et al., 2011; Machado et al., 2020; Avcılar et al., 2024), as well as in Pekin ducks (Onbasilar et al., 2014). For instance, Sahan et al. (2014) showed that eggs from older breeders produced embryos that were 3.7 g heavier and 0.8 cm longer at 18 days of incubation, with these differences increasing to 7.4 g and 1.4 cm at hatch.

Multivariate analysis identified two main axes of variation: one associated with *egg size and nutrient reserves* (initial and final egg weight and embryonic weight) and another related to *effective embryonic growth* (embryonic weight and length). These results suggest that the greater nutritional supply provided by eggs from older breeders supports enhanced embryonic growth (Nangsuay et al., 2011, 2013).

### Post-hatch thermoregulation and performance under thermoneutral conditions

The maintenance of cloacal temperature at approximately 42°C throughout the 42-day experimental period, with no differences between groups, indicates effective homeothermy and the absence of thermal stress (Aluwong et al., 2017; Brugaletta et al., 2022). This stability confirms that environmental conditions within the climatic chamber were adequately controlled to ensure thermal comfort throughout the evaluation period.

Among the thermoregulatory responses evaluated, RR emerged as the most sensitive variable, responding early to differences in maternal origin and postnatal maturation. The observed pattern—higher RR in offspring from older breeders during the first week and in offspring from younger breeders during the second week, followed by a progressive decline from the third week onward—suggests that these fluctuations primarily reflect transient physiological adjustments during the consolidation of thermoregulatory control. In this context, the gradual reduction in RR likely reflects maturation of the thermoregulatory system and a reduced reliance on evaporative heat dissipation mechanisms, particularly given that birds remained under thermoneutral conditions. This progressive, age-related decline in RR is consistent with reports in broiler chickens showing reduced respiratory frequency as birds grow and thermoregulatory efficiency improves (Nascimento et al., 2017).

Surface temperatures (back, eye, and foot) exhibited patterns consistent with peripheral circulatory adjustments for heat dissipation, characterized by an initial increase in dorsal surface temperature followed by a decline, and gradual increases in eye and foot temperatures. These responses reinforce the importance of featherless regions as key sites for heat dissipation (Hernández-Sánchez et al., 2024).

Body weight (BW) increased in a similar and approximately linear manner in both breeder age groups, with no breeder age × week interaction. This finding is consistent with reports indicating that initial weight differences associated with breeder age tend to diminish as growth progresses. Poné et al. (1985) observed that although chicks from older breeders (52 weeks of age) were heavier at hatch, these differences were no longer evident when the chicks reached 44 days of age, with no differences between chicks derived from breeders aged 42 and 52 weeks whereas chicks from younger breeders (27 weeks) showed lower initial body weight and feed intake throughout the evaluation period. Similarly, in the present study, despite maternal age differences between 34 and 63 weeks, growth curves converged over the experimental period.

Factor analysis revealed that birds with higher body weight tended to exhibit lower RR and higher peripheral temperatures, indicating reduced dependence on panting. This pattern can be explained by the greater capacity of heavier birds to redistribute blood flow to peripheral regions such as the feet, eyes, and comb, thereby enhancing sensible heat loss through radiation and convection. This mechanism allows thermal balance to be maintained at a lower physiological cost compared with evaporative heat loss via pulmonary evaporation (Saeed et al., 2019). A second component grouped regional thermal variables (back, head, and wing temperatures), suggesting an axis related to the spatial distribution of body heat.

Discriminant analysis showed that group separation was driven primarily by chronological age (week) rather than breeder age, indicating a limited maternal effect on post-hatch thermoregulation and performance, in agreement with the univariate analyses.

### Egg and body weight as structuring axes of development

Decision tree analyses for both the incubation and post-hatch phases converged in demonstrating that weight—egg weight during embryogenesis and chick body weight after hatch—constitutes the main axis of variation among breeder age × time categories. During the embryonic phase, initial egg weight explained most of the variation associated with breeder age and incubation stage, whereas in the post-hatch period, body weight dominated the segmentation of profiles across weeks of age. Thermoregulatory and morphometric variables, in contrast, modulated only secondary differences along a continuous developmental trajectory. These findings reinforce the importance of egg size and body weight gain as key determinants of broiler development.

### Implications

This study provides integrated evidence that breeder age exerts its most pronounced impact during incubation, whereas its influence on post-hatch performance is relatively modest when broilers are reared under thermally adequate conditions. By combining univariate analyses with multivariate approaches, we demonstrate that eggs from older breeders produce heavier and longer embryos throughout incubation, and that this effect is structured along two main axes: an “egg size and reserve” axis (initial and final egg weight) and an “embryonic growth” axis (embryo weight and length). A key novelty of this work is showing, in an integrated way, that initial egg weight emerges as a pivotal variable capable of discriminating breeder age × incubation day groups, while the remaining embryonic measurements describe a continuous developmental gradient rather than discrete categories. This highlights that egg grading by weight is not merely an operational routine in hatcheries but a critical control point to reduce chick heterogeneity and to safely exploit the developmental potential of eggs from older breeders, which provide greater nutrient supply but are also more susceptible to excessive water loss.

After hatch, our results indicate that maternal age effects become progressively attenuated and that the primary driver of physiological and performance responses is the chronological age of the broilers, rather than breeder age. Although progeny from young and old breeders differed in RR, EyeT and PawT in the early weeks, cloacal temperature remained stable around 42°C, body weight increased linearly, and no breeder age × week interaction was detected. In practical terms, this pattern suggests that, under thermally controlled conditions, the thermoregulatory system matures in a similar manner across progeny from different breeder ages, and that differences in peripheral surface temperatures mainly reflect subtle adjustments in heat dissipation rather than overt thermal stress. Consequently, breeder age should primarily inform egg selection and incubation strategies, whereas in the grow-out phase the emphasis should be on providing uniform thermal comfort and management, without the need for differentiated protocols according to breeder age. Overall, this study supports a “continuum” view linking embryonic and post-hatch development, in which egg weight and body weight account for most of the between-group variation, while thermoregulatory traits serve as indicators of adaptive capacity. These findings offer objective criteria to refine incubation programs and thermal management strategies in broiler production.

## Conclusion

The present study shows that breeder age exerts a pronounced effect on egg characteristics and the early trajectory of embryonic development, whereas its influence on post-hatch growth and thermoregulatory responses is relatively limited when broilers are reared under adequately controlled environmental conditions. During incubation, eggs from older breeders were heavier and produced slightly more developed embryos in terms of mass and length. Multivariate analyses consistently discriminated breeder-age × incubation-day groups along two major axes: one associated with egg size and initial reserves, and another with embryonic growth. Initial egg weight emerged as a central structuring variable, strongly associated with breeder age and accounting for a substantial proportion of between-group variation, while embryonic weight and length described a continuous ontogenetic gradient rather than discrete categories.

In the post-hatch phase, differences between progeny from young and older breeders became progressively subtle and were expressed mainly as minor adjustments in peripheral thermoregulatory traits (notably RR and EyeT), whereas cloacal temperature remained stable and body-weight growth curves were virtually superimposed across breeder ages. Canonical discriminant and decision-tree analyses converged in identifying body weight as the primary organizing variable of physiological profiles during grow-out, with thermal variables contributing to a secondary, finer separation along a continuum of growth and maturation of the thermoregulatory system.

## Data availability

The datasets generated and/or analyzed during the current study are available from the corresponding author on reasonable request.

## AI use disclosure

ChatGPT was used during the preparation of this manuscript to support language refinement, readability, and the preparation of the graphical abstract. All scientific content, data interpretation, and final decisions were made by the authors, who thoroughly reviewed and edited the text and take full responsibility for the manuscript.

## CRediT authorship contribution statement

**Robson Mateus Freitas Silveira:** Writing – original draft, Visualization, Software, Resources, Methodology, Investigation, Formal analysis, Data curation, Conceptualization. **Sergio Luís de Castro Junior:** Writing – review & editing, Validation, Methodology, Investigation, Conceptualization. **Felipe Dilelis:** Writing – review & editing, Visualization, Validation, Resources, Methodology, Investigation, Conceptualization. **Aérica Cirqueira Nazareno:** Writing – review & editing, Visualization, Validation, Methodology, Conceptualization. **Jumara Coelho Ticiano:** Writing – review & editing, Resources, Investigation. **Iran José Oliveira da Silva:** Writing – review & editing, Visualization, Validation, Supervision, Software, Resources, Project administration, Methodology, Investigation, Funding acquisition, Conceptualization.

## Disclosures

The authors declare that they have no conflicts of interest.
